# Predicting immunotherapy response in stage III-IV non-small cell lung cancer using integrated radiomics and clinical features

**DOI:** 10.3389/fonc.2025.1669469

**Published:** 2025-11-07

**Authors:** Na Geng, Zhijun Li, Langlang Deng, Yuxiang Li, Haitao Ma

**Affiliations:** 1 Department of Thoracic Surgery, The Fourth Affiliated Hospital of Soochow University, Suzhou, China; 2 Department of Respiratory Medical, The Second Affiliated Hospital of Bengbu Medical University, Bengbu, China; 3 Department of Rheumatology and Immunology, The Third Affiliated Hospital of Soochow University, Changzhou, China

**Keywords:** radiomics, immunotherapy response, non-small cell lung cancer, nomogram, decision curve analysis

## Abstract

**Objective:**

To develop a combined predictive model based on CT radiomics and clinical features and evaluate its diagnostic value for predicting the efficacy prognosis of immunotherapy in stage III–IV non-small cell lung cancer (NSCLC).

**Methods:**

A retrospective analysis was conducted on 106 patients with stage IIIa–IVB NSCLC who underwent immunotherapy at the Second Affiliated Hospital of Soochow University between December 2018 and December 2023. Patients were divided into two groups based on whether their progression-free survival (PFS) exceeded 12 months. The cohort was randomly split into a training set (75 patients) and a validation set (31 patients) in a 7:3 ratio. Clinical and imaging data were collected, and independent predictive factors were identified through univariate and multivariate logistic regression analysis to construct a clinical feature model. Radiomic features were extracted from contrast-enhanced chest CT images, and LASSO algorithm along with Pearson correlation coefficients were applied to select optimal features and calculate a radiomics score. A combined predictive model integrating clinical independent predictors and radiomic features was developed and visualized as a nomogram. Model performance was assessed by subject work characteristics (ROC) curves and area under the curve (AUC). Clinical utility was assessed via decision curve analysis (DCA), and calibration curves were used to evaluate the nomogram’s predictive accuracy.

**Results:**

Tumor location was an independent predictor of immunotherapy efficacy and formed the clinical model. Twelve contrast-enhanced CT radiomic features comprised the radiomics model. The combined model (clinical + radiomic) demonstrated superior diagnostic performance: training set AUCs (clinical: 0.705, radiomics: 0.835, combined: 0.896);validation set AUCs (clinical: 0.691, radiomics: 0.833, combined: 0.863). The combined model’s AUC was significantly higher than either submodel alone in both sets. DCA confirmed its highest net clinical benefit, and calibration curves indicated good accuracy.

**Conclusion:**

This study developed a predictive model based on clinical and radiomic features for assessing immunotherapy efficacy in NSCLC. The model demonstrated excellent performance, suggesting its potential as a clinical decision-support tool for prognosis prediction and treatment planning in NSCLC immunotherapy.

## 1 Introduction

The advent of immune checkpoint inhibitors (ICIs) has revolutionized the treatment paradigm for advanced non-small cell lung cancer (NSCLC), with approximately 20-30% of patients achieving durable responses (1). However, the current gold-standard biomarker PD-L1 expression exhibits limited predictive accuracy (objective response rate concordance: 45-60%) (2, 3), while emerging biomarkers like tumor mutational burden (TMB) face challenges in clinical standardization (4–6). This predictive uncertainty leads to non-negligible rates of premature treatment discontinuation (34.7% in real-world studies (7)) and unnecessary immune-related adverse events (irAEs),highlighting the urgent need for more robust stratification tools. Radiomics, as a non-invasive approach decoding tumor heterogeneity through high-throughput imaging feature analysis, has demonstrated unique advantages in ICI response prediction. Recent studies revealed that CT-based radiomic signatures could reflect tumor microenvironment characteristics (e.g., CD8+ T-cell infiltration (8)) and predict progression-free survival (PFS) with AUCs of 0.71-0.79 (9, 10).By extracting quantitative radiomics features from patient CT images and computing the radiomics signature, combined with selecting predictive clinical indicators from patient medical records, we developed and validated a Combined Forecasting Model (CFM) to predict treatment response to immunotherapy in non-small cell lung cancer (NSCLC) patients.

## 2 Materials and methods

### 2.1 General information

This study is a retrospective, multicenter investigation that collected clinical and imaging data from patients with advanced NSCLC who received ICIs treatment at two domestic centers: the Second Affiliated Hospital of Soochow University (Center 1) and the First Affiliated Hospital of Soochow University (Center 2) between December 2018 and December 2023.Baseline clinical data and computed tomography (CT) imaging were collected for all participants,. This study was conducted in accordance with the ethical principles of the Declaration of Helsinki (2013 revision) and received approval from the Ethics Committee of the First Affiliated Hospital of Soochow University and the Second Affiliated Hospital of Soochow University. Inclusion Criteria: ①Pathologically confirmed stage IIIa-IVb NSCLC according to the 9th edition of the TNM system (10) ②Treatment with at least two cycles of either anti-PD-1/PD-L1 monotherapy or combination therapy of immunotherapy with platinum-based doublet chemotherapy. Patients who received concurrent or sequential radiotherapy, targeted therapy, or any other anticancer regimens were excluded. ③Availability of contrast-enhanced CT scans obtained within 2 months prior to immunotherapy initiation ④Complete clinical documentation;Exclusion Criteria: ①Primary tumor undetectable or unsegmentable on chest CT imaging, or poor image quality ②Receipt of any additional anticancer therapy beyond specified immunotherapy or chemotherapy regimens ③Presence of active autoimmune diseases ④Loss to follow-up or incomplete postoperative surveillance records.

### 2.2 Indicator collection

Clinical data collected included patient demographics (age, sex, smoking history), tumor characteristics (non-small cell lung cancer histologic subtype, PD-L1 expression level, tumor stage, location), serum fibrinogen levels, and enhanced CT imaging findings. The administered anti-PD-1/PD-L1 monoclonal antibodies included Camrelizumab, Pembrolizumab, Sintilimab, Tislelizumab, and Toripalimab (see **Table 1** for details).Treatment response was assessed per RECIST v1.1, classifying outcomes as progressive disease (PD), stable disease (SD), partial response (PR), or complete response (CR) using electronic medical records. The primary endpoint was progression-free survival (PFS), defined as the time from immunotherapy initiation until disease progression, death from any cause, or final follow-up. Patients were stratified into two groups based on whether their PFS exceeded 12 months. A PFS threshold of 12 months was chosen to define the durable clinical benefit (DCB) group, as it is a commonly used surrogate endpoint in immuno-oncology studies to reflect sustained treatment efficacy and is clinically meaningful for prognosis assessment.

**Table 1:** Baseline characteristics of patients in the training and external validation cohorts.

Characteristict	Training cohort (n=75)^1^	External validation cohort (n=34)	^1^P-value
Age (years)	70.0 (8.8)	72 (9.9)	0.315
Sex			0.684
Female	11 (15%)	4 (12%)	
Male	64 (85%)	30 (88%)	
PD-L1 Expression			0.017
<1%	9 (12%)	9 (26%)	
1-49%	36 (48%)	20 (59%)	
≥50%	30 (40%)	5 (15%)	
Tumor Location			0.826
Central	37 (49%)	16 (47%)	
Peripheral	38 (51%)	18 (53%)	
Cancer Type			0.576
Squamous	44 (59%)	18 (53%)	
Adenocarcinoma	31 (41%)	16 (47%)	
T Stage			0.844
3A	6 (8.0%)	3 (9%)	
3B	17 (23%)	9 (26%)	
3C	8 (11%)	3 (9%)	
4A	25 (33%)	8 (24%)	
4B	19 (25%)	11 (32%)	
Immunotherapy Drug			0.221
Camrelizumab	10 (13%)	9 (26%)	
Pembrolizumab	19 (25%)	8 (24%)	
Sintilimab	27 (36%)	12 (35%)	
Tislelizumab	18 (24%)	4 (12%)	
Toripalimab	1 (1.3%)	1 (3%)	
Treatment Regimen			0.773
Imm.	15 (20%)	6 (18%)	
Imm. + Chemo.	60 (80%)	28 (82%)	
Smoking Status			0.251
Non-smoker	33 (44%)	11 (32%)	
Smoker	42 (56%)	23 (68%)	
Fibrinogen	4.23 (1.18)	3.63 (0.98)	0.728

^1^Data presented as n (%) or mean (SD).

### 2.3 Analysis and extraction of radiomics features

Enhanced CT images in DICOM format were retrieved from the institutional PACS system and anonymized. The contrast-enhanced CT images were reviewed, and tumor lesions were manually segmented slice-by-slice on lung window settings (width: 1500 HU; level: -600 HU) using 3D Slicer software (v4.11) to define volumetric regions of interest (VOIs). The use of contrast-enhanced images facilitated more accurate delineation of tumor boundaries, particularly in distinguishing the tumor from adjacent vessels and atelectatic lung tissue. A total of 1,036 radiomics features were extracted from 3D VOIs using the PyRadiomics package (v3.0.1) in Python, encompassing four feature classes:1.First-order statistics (e.g., intensity histogram metrics)2.Shape-based features (3D orphological descriptors)3.Texture features: Gray-Level Co-occurrence Matrix (GLCM): Correlation, Energy Gray-Level Run- Length Matrix (GLRLM): Long Run Emphasis (LRE) Gray-Level Size Zone Matrix (GLSZM): Size Zone Non-Uniformity 4.Higher-order features (filtered image approximations).To evaluate the reproducibility of radiomic feature extraction, both inter-observer and intra-observer reliability were assessed. For inter-observer reliability, volumetric segmentations were performed independently by two radiologists on a randomly selected subset of 30 patients. For intra-observer reliability, the primary radiologist repeated the segmentations on the same subset after a four-week interval to minimize memory bias. The intraclass correlation coefficient (ICC) was calculated for both assessments. Only features exhibiting excellent reproducibility (ICC > 0.75) in both evaluations were retained for subsequent analysis. Features with ICC >0.75 (11, 12) were retained, indicating excellent agreement according to established criteria.

### 2.4 Construction of clinical characterization model, CT imaging histology model

Using the “Pyradiomics” package in Python to extract radiomics features, we first conduct Z-score normalization on the extracted radiomics features. Then, we perform dimensionality reduction on the radiomics data using mRMR and LASSO. We use Logistic Regression(LR)as the classifier to build the radiomics model. Moreover, we adopt 10-fold cross-validation to enhance the stability of the model. Univariate logistic regression analysis was used to screen out the predictors. Subsequently, multivariate logistic regression analysis was used for the clinical indicators with statistical differences in univariate analysis to screen out the independent predictors, and a clinical model was constructed based on this.

### 2.5 Construction and evaluation of the CFM

The independent clinical predictors and the radiomics score (Rad-score) were integrated into a final multivariate logistic regression analysis to construct the Combined Forecasting Model (CFM). A nomogram was generated to visualize this model. The performance of all models was evaluated using several metrics. Receiver Operating Characteristic (ROC) curves were used to assess discriminative ability, calibration curves were used to evaluate model reliability, and Decision Curve Analysis (DCA) was used to assess the net clinical benefit and clinical utility the independent predictors and radiomics scores were included in the multivariate logistic regression analysis, and a combined model was constructed based on this, and a nomogram was drawn. The ROC curve was used to evaluate CFM, the calibration curve was used to evaluate the reliability of the model, and the DCA was used to evaluate the clinical efficacy of the radiomics model and CFM.

## 3 Results

### 3.1 Baseline characteristics of patients in the training and external validation cohorts

No significant differences in baseline characteristics existed between the training and external test sets, as confirmed by independent samples t-tests/Mann-Whitney U tests for continuous variables and chi-square tests for categorical variables (**Table 1**). The balanced distribution of clinical features across both cohorts supports the validity of the external validation approach and ensures that performance differences reflect true model efficacy rather than cohort disparities.

### 3.2 Rad-scores and their predictive performance

**Figure 1:**
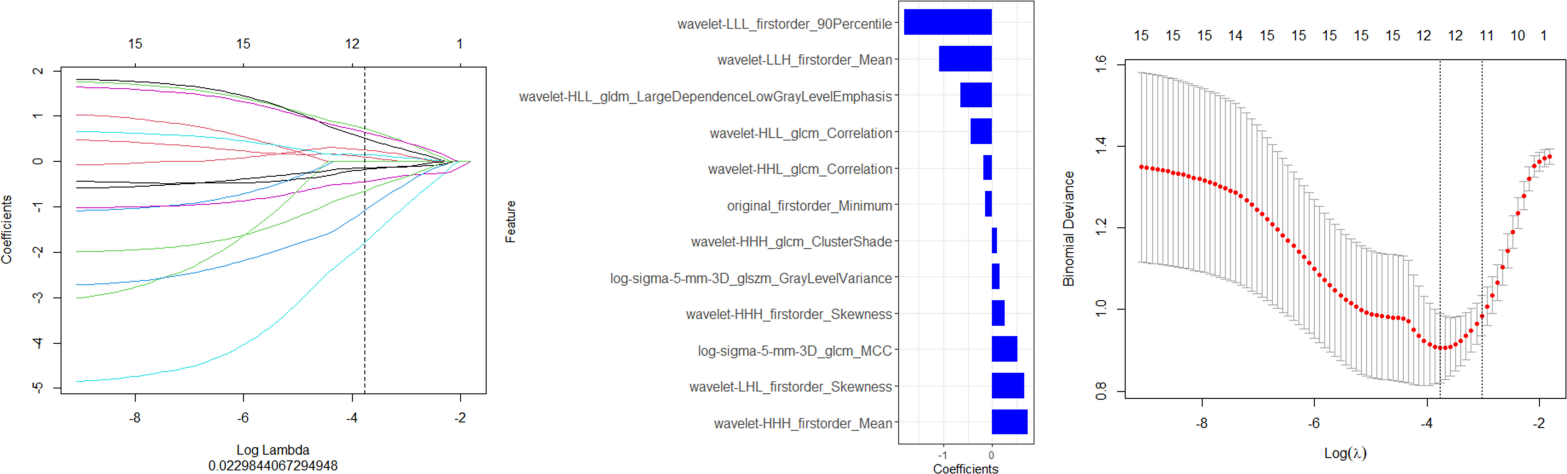
Screening of the radiomics features in the training set via LASSO regression. **(A)** Features maintaining non-zero coefficients at the **(A)** 1se threshold (vertical dashed line) in LASSO regression analysis." **(B)** The final feature subset with non-zero coefficients selected by LASSO regression at the optimal penalty parameter (A. 1se) **(C)** The optimal regularization parameter (A. 1se) was determined through cross-validation, as indicated by the vertical dashed line on the right.

**Figure 2:**
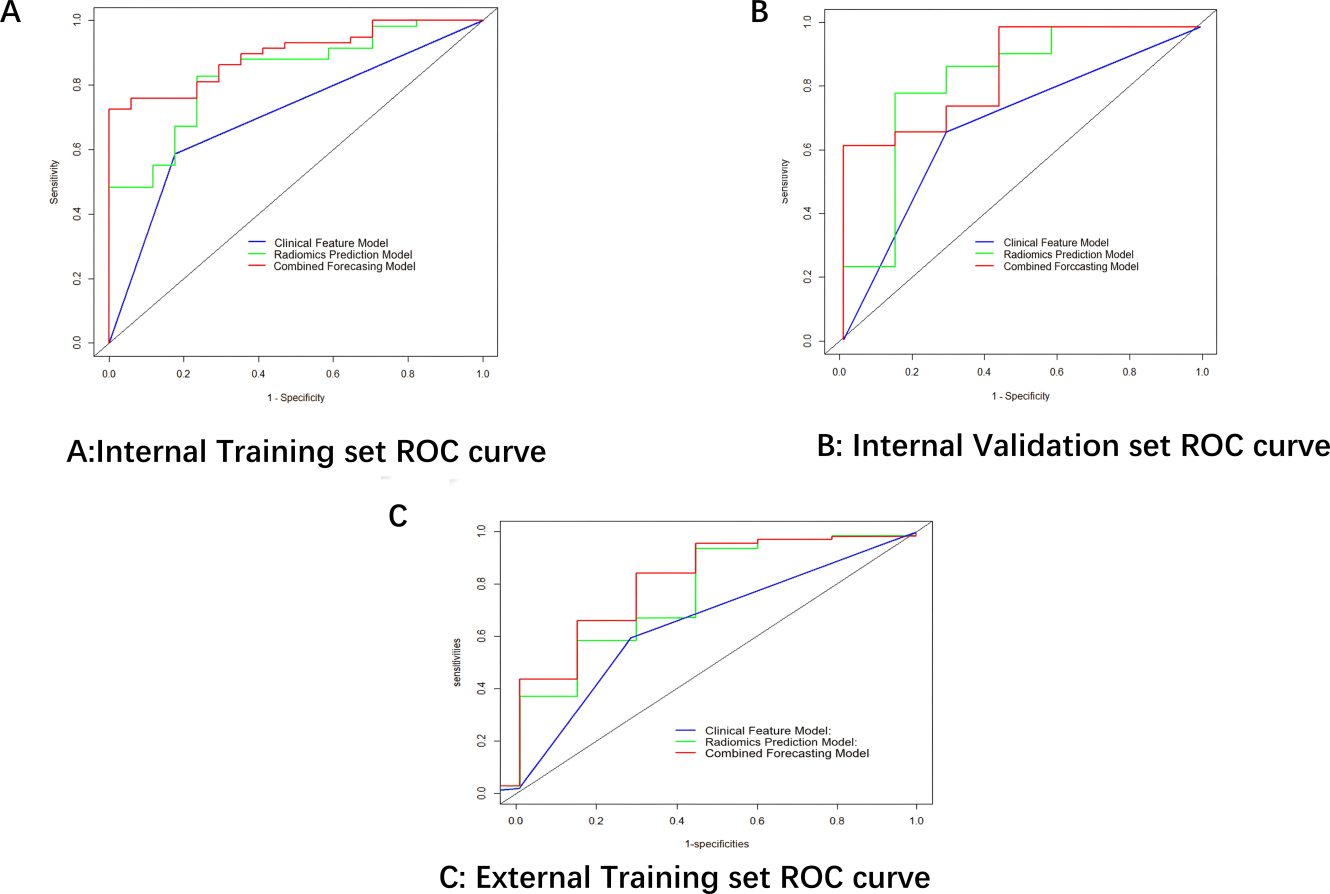
ROC curves comparing model performance across datasets.

Finally,12 radiomic features with non-zero coefficients were obtained from the 1036 radiomic features in the training set using the LASSO method. (**Figure 1**) The radiomics prediction models for the training and validation sets achieved AUC values of 0.835 and 0.833 under the ROC curve, respectively. The accuracy rates were 0.813 and 0.806,respectively, and the sensitivity rates were 0.828 and 0.792, respectively. (**Figure 2**) These results indicate that the radiomics model demonstrated robust performance in predicting immunotherapy efficacy. The radiomics features of the above-mentioned radiomics model were linearly combined and weighted by their respective coefficient AUC = 0.896 (0.825-0.966) to calculate the rad-scores.

### 3.3 Construction of the clinical model

Collecting clinical data on all patients. Univariate and multifactorial logistic regression analyses showed that tumor location: peripheral or central was of predictive value (**Table 2**).

**Table 2:** Univariate and multivariate logistic regression analysis of predictive factors.

Variable	Univariate logistic regression analysis OR (95%CI)	p-value^2^	Multivariate logistic regression analysis OR (95%CI)	p-value^2^
Age (years)	2.242 (0.569-8.832)	0.143		
Sex	2.242 (0.569-8.832)	0.249		
PD-L1 Expression	1.897 (0.837-4.302)	0.125		
Tumor Location	0.151 (0.039-0.585)	0.006	0.127 (0.025-0.653)	0.013
Cancer Type	0.739 (0.249-2.194)	0.586		
Stage	1.596 (0.997-2.554)	0.052		
Treatment Regimen	0.462 (0.093-2.284)	0.343		
Smoking	1.594 (0.538-4.723)	0.4		
Fibrinogen	0.820 (0.524-1.284)	0.386	3.63 (0.98)	
rad_score	3.305 (1.662-6.569)	<0.001		<0.001

^1^Data presented as n (%) or mean (SD).

### 3.4 Performance and evaluation of the Combined Forecasting Model

**Figure 3:**
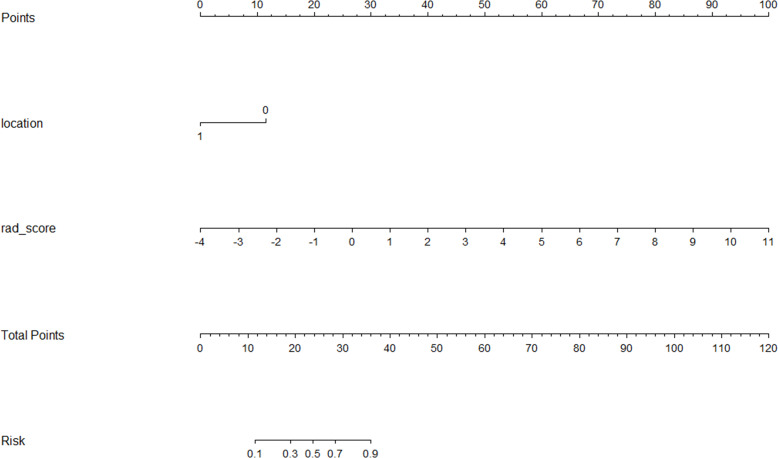
Nomogram of the comprehensive forecasting model.

**Figure 4:**
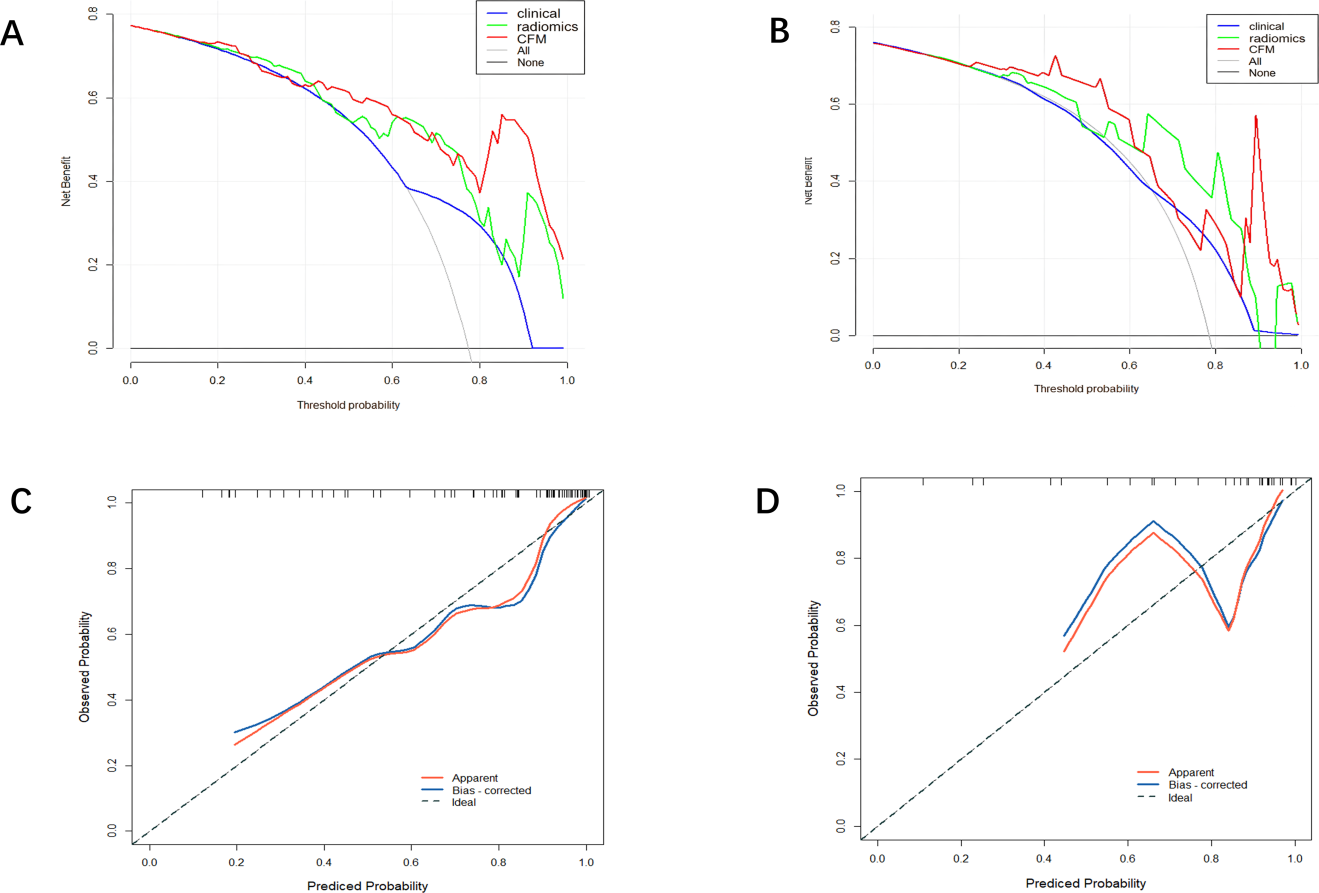
**(A)** Training set DCA curve **(B)** Validation set DCA curve **(C)** Calibration curve of the comprehensive forecasting model in the training set. **(D)** Calibration curve of the comprehensive forecasting model in the test set.

**Figure 5:**
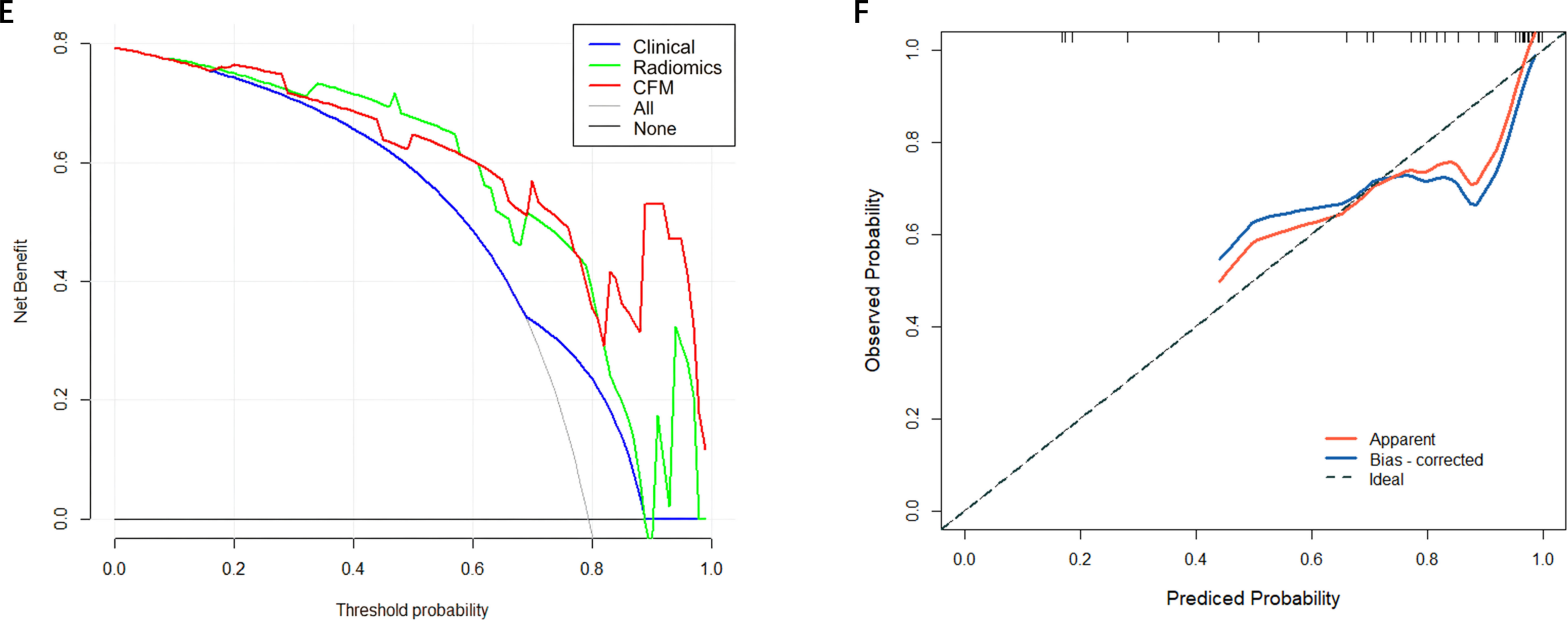
**(E)** External Validation DCA curve **(F)** Calibration curve of the comprehensive forecasting in the External Validation.

A CFM was established by incorporating the Rad-score and the independent clinical factor (tumor location) into a logistic regression model, which was visualized as a nomogram (**Figure 3**). The performance of the CFM was superior to the individual models. As shown in **Table 3**, the CFM achieved an AUC of 0.902 (95% CI: 0.833-0.970) in the internal training cohort and 0.863 (95% CI: 0.813-1.000) in the internal validation cohort. The model’s high performance was maintained in the independent external validation cohort (AUC = 0.863, 95% CI: 0.813-1.000), underscoring its robustness and generalizability. To quantitatively assess the model’s performance and generalizability, DeLong tests were conducted to compare the AUCs among the three models within each cohort. In the training cohort, the combined model significantly outperformed the clinical model (p < 0.001). However, no significant differences were found between the combined model and the radiomics model (p = 0.095), nor among any of the three models in both the internal validation (all p > 0.30) and external validation cohorts (all p > 0.07). These results indicate that the combined model achieves robust and stable predictive performance without overfitting, generalizing well to independent patient cohorts. Detailed results of the pairwise comparisons are provided in **Supplementary Figure 1**. Decision Curve Analysis (DCA) demonstrated that the CFM provided the highest net clinical benefit across a wide range of threshold probabilities compared to the clinical-only or radiomics-only models in all cohorts (**Figure 4**). Furthermore, the calibration curves indicated good agreement between the model’s predicted probabilities and the actual observed outcomes in both the training and validation datasets, confirming the model’s reliability (**Figure 5**).

**Table 3:** Performance metrics of clinical, radiomics and combined models across datasets.

Model	Dataset	AUC (95% CI)	Accuracy	Sensitivity	Specificity
Clinical Model	Internal Training Set	0.705 (0.592-0.818)	0.64	0.586	0.824
	Internal Validation Set	0.691 (0.486-0.895)	0.677	0.667	0.714
	External Validation Set	0.653 (0.450-0.857)	0.618	0.593	0.714
	Internal Training Set	0.835 (0.733-0.937)	0.813	0.828	0.765
Radiomics Model	Internal Validation Set	0.833 (0.624-1.000)	0.806	0.792	0.857
	External Validation Set	0.831 (0.641-1.000)	0.912	1	0.571
	Internal Training Set	0.896 (0.825-0.966)	0.787	0.724	1
Combined Model	Internal Validation Set	0.863 (0.713-1.000)	0.71	0.625	1
	External Validation Set	0.884 (0.757-1.000)	0.735	0.667	1

## 4 Discussion

This study integrated clinical characteristics and CT radiomics features of patients with stage III-IV non-small cell lung cancer (NSCLC) receiving immunotherapy to develop an integrated diagnostic model. This study first explored independent predictors of immunotherapy efficacy in the clinical setting and found that central tumor location was adverse prognostic outcomes in NSCLC. Previous studies (13, 14) have shown that in terms of microenvironment, central-type tumors have 2.8-fold more TGF-β expression than peripheral-type, CD8+ T-cell density is decreased by 40% under multiple fluorescence, and central-type lung cancers have significantly worse (15) OS than peripheral-type lung cancers; there are also previous studies (16) that emphasized the peritumor texture-entropy imaging histological features of central-type lung cancers compared with the peripheral-type lung cancers. Based on the above independent influences on the efficacy of immunotherapy, the present study firstly modeled the clinical characteristics of prognosis. Utilizing data from the Second Affiliated Hospital of Soochow University, a cohort of 106 patients was included. The constructed integrated diagnostic model demonstrated (17)superior diagnostic efficacy, achieving areas under the AUC of 0.902 and 0.863 in the training and validation sets. The nomogram developed in this study serves as an intuitive tool with potential for integration into clinical decision support systems (CDSS). In future clinical practice, prior to initiating immunotherapy, clinicians could input the patient’s tumor location and CT-based Rad-score to obtain an individualized probability of treatment response. This approach would help identify patients likely to derive suboptimal benefit, enabling consideration of alternative treatment strategies or more intensive monitoring, ultimately advancing toward precision immunotherapy for lung cancer. Furthermore, grayscale normalization techniques (18) were applied during preprocessing to mitigate image variations attributable to differences in imaging acquisition parameters. Comparative analysis using ROC curves evaluated the clinical feature model, CT radiomics model, and the integrated diagnostic model. The results revealed that the AUC of the integrated model was significantly higher than the other two models in both the training and validation sets, indicating its robust diagnostic performance and favorable generalizability (19).DCA demonstrated that the integrated diagnostic model yielded the highest net clinical benefit across both the training and validation cohorts compared to the clinical feature model and the CT radiomics model. Calibration curves indicated good agreement between predicted and observed outcomes for the integrated model, although variations in performance across different predicted probability ranges were noted among all models. This study has several limitations that should be considered. First, despite being a two-center study, its retrospective design may still introduce selection bias (20). To mitigate this, we applied strict, consistent inclusion and exclusion criteria across both participating centers. Second, the sample size, particularly for the external validation cohort (n=34), remains relatively small (21, 22), which may affect the stability of the estimates. Future large-sample studies needed to confirm our findings. we acknowledge that the sample size, particularly of the external validation cohort, is relatively modest. While larger-scale prospective studies are certainly needed to further solidify our findings, a key strength of our work is the very inclusion of an independent external validation set. The model’s excellent and stable performance in this cohort (AUC: 0.863) provides strong preliminary evidence of its generalizability, addressing a common limitation in many radiomics studies. Third, heterogeneity in immunotherapy regimens exists. Although our univariate analysis showed that the specific treatment regimen was not a significant predictor, the eterogeneity in drugs and combinations (e.g., mono-immunotherapy vs. chemo-immunotherapy) remains a limitation. We subsequently performed a *post hoc* exploratory analysis by stratifying patients according to treatment regimen (immunotherapy monotherapy versus combined immunotherapy-chemotherapy). The combined model maintained robust discriminatory capacity across all subgroups (all AUCs > 0.80); however, formal statistical comparisons were not conducted due to limited sample sizes within the subgroups. Future studies with larger, more homogeneous cohorts could further refine the model’s applicability. Fourth, manual segmentation inherently carries a risk of inter-observer variability. We minimized this by having segmentations performed by an experienced radiologist following a standardized protocol and by rigorously assessing feature reproducibility (ICC > 0.75). Fifth, our study currently lacks delta-radiomics (23–25) from longitudinal imaging, which represents a promising future direction to capture dynamic changes in tumor biology during therapy and potentially further enhance predictive performance (26, 27).

## Data Availability

The raw data supporting the conclusions of this article will be made available by the authors, without undue reservation.
