# Supplementary material for: Predicting immunotherapy response in stage III-IV non-small cell lung cancer using integrated radiomics and clinical features
Source: Front Oncol. 2025 Nov 7;15:1669469. doi: 10.3389/fonc.2025.1669469 (PMC12634321; doi:10.3389/fonc.2025.1669469)
Supplement: Supplementary file 1 [file DataSheet1.docx]

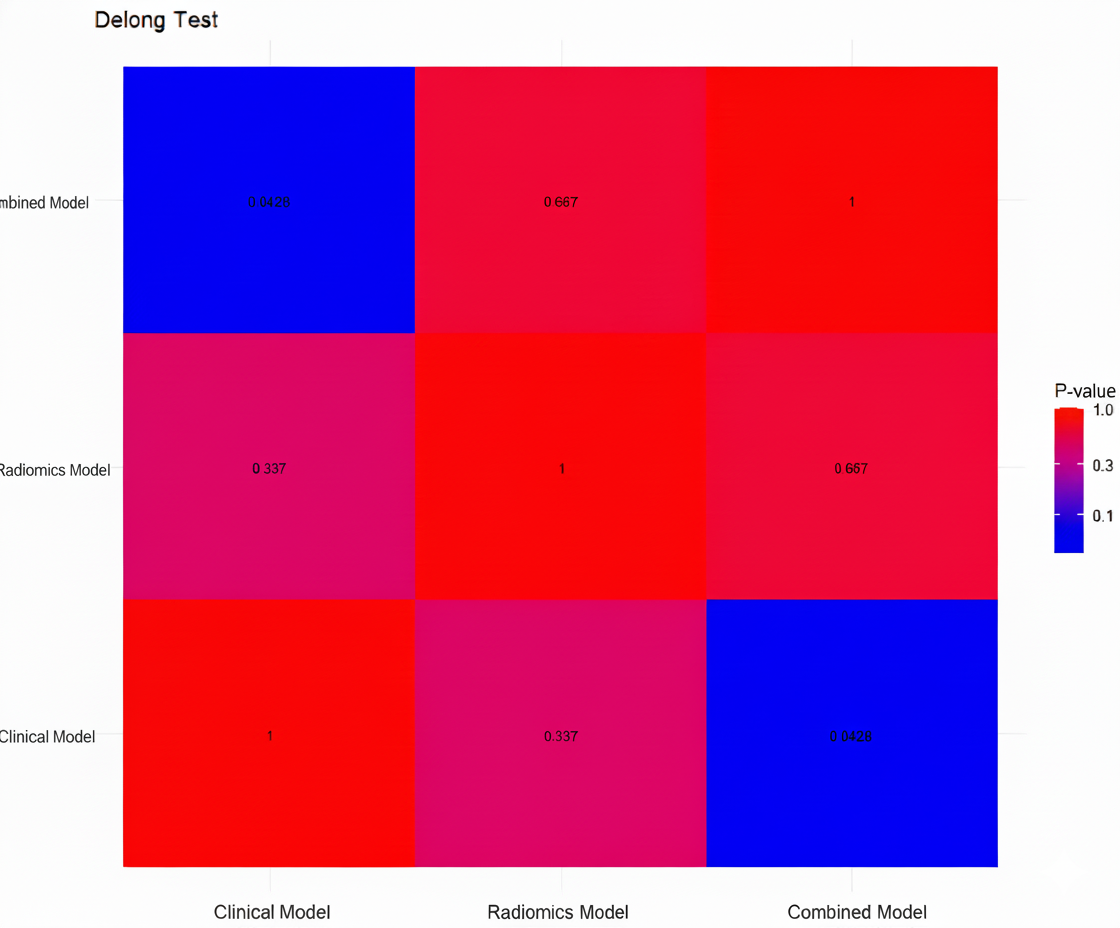


Internal training set DeLong tests


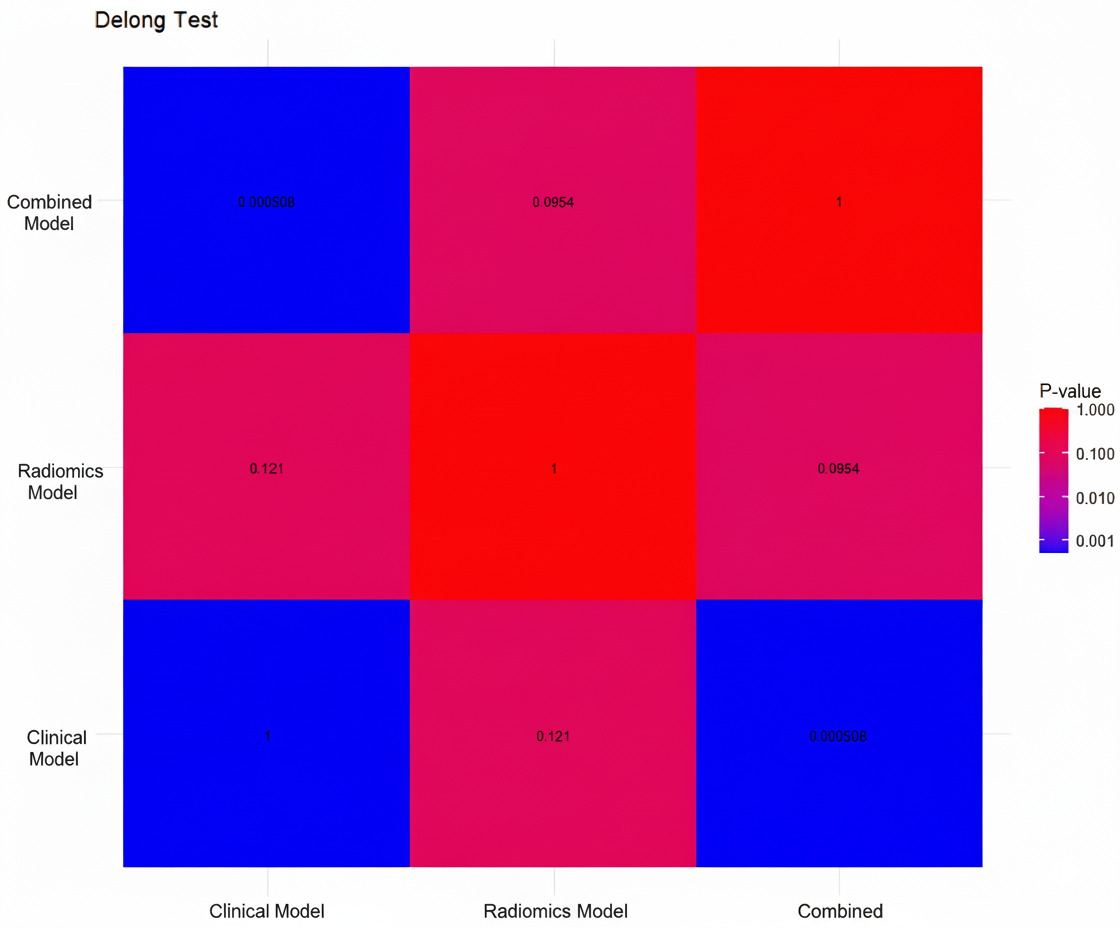


Internal validation set DeLong tests

DeLong tests


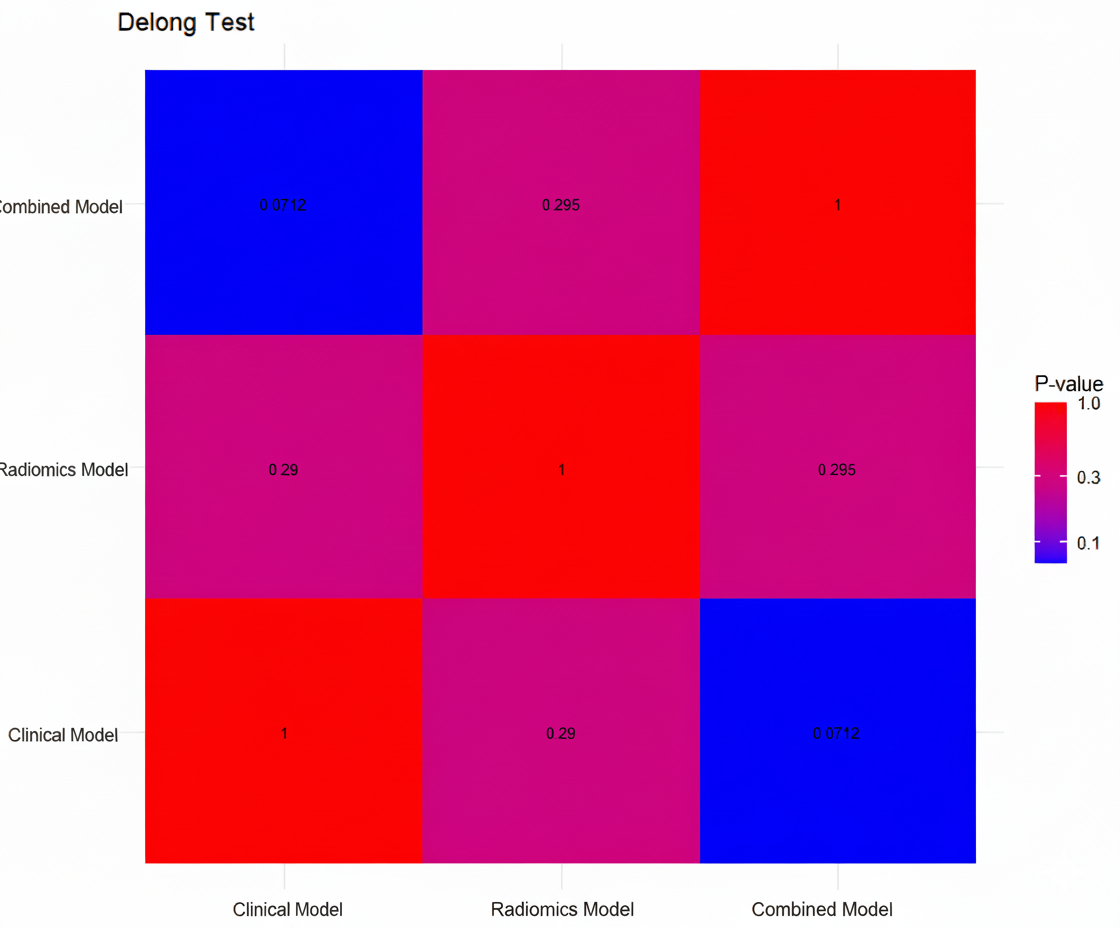


External Validation DeLong tests
